# Examining perceptions of a telemedicine network for pediatric emergency medicine: a mixed-methods pilot study

**DOI:** 10.3389/fdgth.2023.1181059

**Published:** 2023-05-26

**Authors:** Lidia A. Mateus, Madelyn P. Law, Asif Raza Khowaja, Elaina Orlando, Alexander Pace, Madan Roy, Christopher Sulowski

**Affiliations:** ^1^ Faculty of Applied Health Sciences, Brock University, St. Catharines, ON, Canada; ^2^ Research Department, Niagara Health, St. Catharines, ON, Canada; ^3^ Department of Pediatrics, Niagara Health, St. Catharines, ON, Canada; ^4^ Department of Pediatrics, McMaster Children's Hospital, Hamilton, ON, Canada; ^5^ Department of Pediatrics, Division of Pediatric Emergency Medicine, McMaster Children's Hospital, Hamilton, ON, Canada

**Keywords:** telemedicine, tele-resuscitation, pediatrics, critical care, emergency medicine

## Abstract

**Background:**

Use of telemedicine for healthcare delivery in the emergency department can increase access to specialized care for pediatric patients without direct access to a children's hospital. Currently, telemedicine is underused in this setting.

**Objectives:**

This pilot research project aimed to evaluate the perceived effectiveness of a telemedicine program in delivering care to critically ill pediatric patients in the emergency department by exploring the experiences of parents/caregivers and physicians.

**Methods:**

Sequential explanatory mixed methods were employed, in which quantitative methods of inquiry were followed by qualitative methods. Data were collected through a post-used survey for physicians, followed by semi-structured interviews with physicians and parents/guardians of children treated through the program. Descriptive statistics were used to analyze the survey data. Reflexive thematic analysis was used to analyze interview data.

**Results:**

The findings describe positive perceptions of telemedicine for emergency department pediatric care, as well as barriers and facilitators to its use. The research also discusses implications for practice and recommendations for overcoming barriers and supporting facilitators when implementing telemedicine programming.

**Conclusion:**

The findings suggest that a telemedicine program has utility and acceptance among parents/caregivers and physicians for the treatment of critically ill pediatric patients in the emergency department. Benefits recognized and valued by both parents/caregivers and physicians include rapid connection to sub-specialized care and enhanced communication between remote and local physicians. Sample size and response rate are key limitations of the study.

## Introduction

In the field of emergency medicine, pediatric patients are a vulnerable population, often requiring subspecialized care to ensure optimal patient outcomes (1). The level of specialization required to treat pediatric emergency Canadian Triage and Acuity Scale (CTAS) levels 1 and 2 cases is typically only found in children's hospitals rather than in community hospitals (2). CTAS 1 refers to conditions requiring resuscitation and CTAS 2 refers emergent conditions. As a result, access to subspecialized care is limited for families with children living in areas without direct access to children's hospitals. In such communities, health disparities are common and may include higher rates of chronic illness and emergency department (ED) overutilization. This leads to a greater burden on patients, care providers, and the health care system (3). Given this knowledge, it is necessary to find methods to increase access to subspecialized care for critically ill pediatric patients in peripherally located community hospitals.

A southern Ontario community hospital system, the “local hospital”, partnered with a tertiary care children's hospital, the “remote hospital”, to develop a telemedicine program for critically ill pediatric patients. Pediatric Telemedicine Connecting Hospitals (Peds-TECH) facilitates telemedicine-based resuscitation for infants/children that present at the local hospital through real-time two-way audio-visual consultation. Peds-TECH functions through a “hub-and-spoke” model with five hospital sites within the local hospital and one tertiary care children's hospital as the remote hospital, averaging two to three cases per month.

This pilot study aimed to explore care provider and user perceptions about using telemedicine and their experience of telemedicine utilization in the ED, as well as to identify potential barriers and facilitators of use of Peds-TECH. This pilot will inform feasibility of the chosen methods for a larger scale study at this site and inform the potential for this innovative approach to be spread to other similar sites.

The research questions were:

1. What are physicians' perceptions of care delivery through telemedicine?
  a. What are the barriers and facilitators to telemedicine utilization?
2. What are parents'/caregivers’’ experiences of their child receiving care through telemedicine?

## Methods

### Study design

The study employed a sequential explanatory mixed-methods design, in which the quantitative data were collected first to inform the design of qualitative inquiry (4). A similar approach has been used elsewhere for health technology assessment and was relevant to address the research questions for study (5–7).

### Population

The study population consisted of ED physicians from the local and remote hospitals, as well as parents and caregivers of children treated through Peds-TECH. Physician participants were contacted via email and invited to participate in the study. Inclusion criteria consisted of (a) being a physician in the ED of a participating hospital, (b) having directly interacted with Peds-TECH at least once, and (c) being able to communicate in English. Parents/caregivers participants were contacted by a designate affiliated with the local hospital. The designate introduced the study, provided consent forms, and received verbal consent to be contacted by the researcher. Inclusion criteria consisted of (a) having a child who was part of an interaction with the Peds-TECH program at least once and (b) being able to communicate in English.

### Data collection

Quantitative data was collected from physicians through an online survey. The purpose of the survey in this study was to guide the qualitative inquiry by ensuring that the questions in the interview addressed relevant elements physicians' experiences. Survey questions were developed in collaboration with ED physician content experts and guided by the literature in adult populations, particularly the survey questions outlined in the Telemedicine for Trauma Resuscitation project manual (8). Questions included an open-ended case description, discussion of infrastructure, team interactions, and patient care in relation to telemedicine use. Each of these sections included questions on a 5-point Likert scale as well as an open-ended question. The survey was reliable as noted by a Cronbach's alpha value of 0.928, denoting a high level of internal consistency (9). Following this, semi-structured interviews were conducted with both participant groups. The content of the physician interviews was developed following an analysis of the survey results. The parent/caregiver interview guide was developed in collaboration with an ED physician content expert and piloted with an experienced nurse. Topics of discussion included perceptions of the use of technology, if and how they were engaged during the care process, and aspects of the interactions that could have been improved. All interviews were conducted and recorded virtually through Microsoft Teams. Interviews were initially automatically transcribed through Microsoft Teams and verified by the researcher for accuracy. Physician interviews ranged from 14 to 43 min in length, while parent/caregiver interviews ranged from 12 to 21 min. The survey and interview guides can be found in Supplementary Appendix A.

### Data analysis

Likert scale data were analyzed using descriptive statistics by way of frequency, mean, standard deviation, and confidence interval (10). In accordance with Boone and Boone (10), mean was chosen as the measure of central tendency because the survey employed Likert scales, which are groups of Likert-type questions considered together to provide a measure of attitude on a single trait. Pearson correlation coefficient was calculated within the SPSS program (Version 28.0.1.0) to measure the strength and direction of the linear relationship between infrastructure and patient care sections on the Likert scale. The survey was reliable as noted by a Cronbach's alpha value of 0.928, denoting a high level of internal consistency (9). Results of the survey aided in developing questions and topics explored in the interviews. Qualitative data were analyzed using the NVivo software following the six-phase process that accompanies reflexive thematic analysis. Analysis consisted of familiarization, open inductive coding, theme development, refining and naming themes, and representing finding and interpretations (11).

### Sample size

A total of 31 participants were enrolled in the study. Eleven of the 38 physician participants invited completed a survey, 4 from the local hospital and 7 from the remote hospital (28.9% response rate). Fifteen of the 29 physicians invited engaged in interviews, with 13 from the local hospital and 2 from the remote hospital (51% response rate). Five of 40 parent/caregiver participants invited engaged in interviews (12.5% response rate). Despite recruitment challenges in the parent/caregiver group, consistent themes were present throughout the interviews and saturation was achieved.

### Ethics approval

The study was approved by the [blocked for peer review] Research Ethics Board and [blocked for peer review] Research Ethics Board.

## Results

### Physician survey

Surveys were sent to the physicians following each telemedicine encounter, facilitating monitoring of quality improvement efforts and inquiry into key elements of the program. There was variability in responses as seen in Table 1. Participants responded positively to the use of Peds-TECH across categories of Infrastructure, Interactions, and Patient Care. As demonstrated in Table 1, the infrastructure of the program functioned adequately to meet the needs of users. The majority of participants either agreed or strongly agreed that the quality of audio (90.9%) and video (81.9%) was adequate. The majority of participants agreed or strongly agreed that there was an appropriate amount of closed-loop communications (72.8%). Additionally, most participants agreed or strongly agreed that members of the team understood their role (81.9%). Finally, within the Patient Care category, 63.7% of participants agreed that Peds-TECH was more effective than a phone call for delivering patient care. This result appeared to be contingent on the perception that Peds-TECH was used appropriately; participants who disagreed with the statement noted that their consultation could have been delivered by phone or that video was not necessary.

**Table 1:** Infrastructure, interactions, and patient care.

Question	Strongly agree [4]	Agree [3]	Disagree [2]	Strongly disagree [1]	Neutral [0]	Mean	Standard deviation	Confidence interval (95%)
Infrastructure: The quality of the audio was adequate	4 (36.4%)	6 (54.5%)	0 (0%)	1 (9.1%)	0 (0%)	3.18 Agree	±2.76	3.18 ± 1.631
Infrastructure: The quality of the video was adequate	4 (36.4%)	5 (45.5%)	0 (0%)	1 (9.1%)	1 (9.1%)	2.91 Agree	±2.66	2.91 ± 1.572
Interactions: There was an appropriate amount of closed loop communications	5 (45.5%)	3 (27.3%)	0 (0%)	1 (9.1%)	2 (18.2%)	2.73 Agree	±2.66	2.73 ± 1.572
Interactions: Members of the team understood their roles	5 (45.5%)	4 (36.4%)	0 (0%)	1 (9.1%)	1 (9.1%)	3 Agree	±2.76	3 ± 1.631
Patient Care: The use of OTN (Ontario Telemedicine Network) was more effective than a phone call	4 (36.4%)	3 (27.3%)	2 (18.2%)	1 (9.1%)	1 (9.1%)	2.73 Agree	±2.52	2.73 ± 1.489

**Figure 1:**
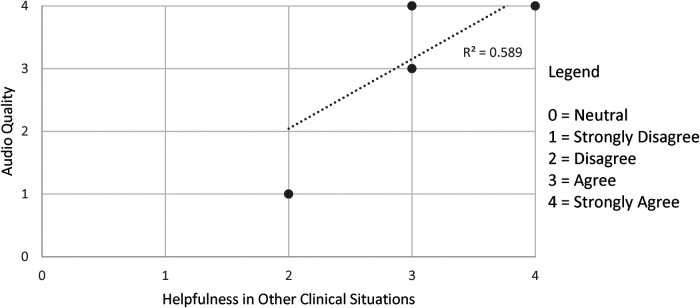
Audio quality and helpfulness in clinical situations. *Note.* The data displayed were from the statements “The quality of the audio was adequate” and “I believe that this type of telemedicine approach would be helpful in other clinical situations”. There were 11 pairs data points submitted, repeated responses were not displayed. *r* = 0.880, *α* = 0.05.

**Figure 2:**
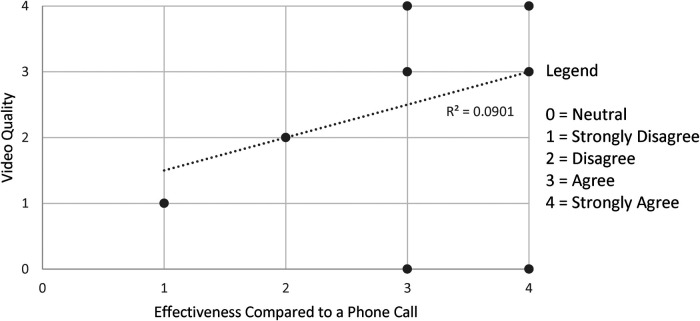
Video quality and effectiveness compared to a phone consult. *Note.* The data displayed were from the statements “The quality of the video was adequate” and “The use of OTN was more effective than a phone call (e.g. direct telephone consultation)”. There were 11 pairs data points submitted, repeated responses were not displayed. *r* = 0.783, *α* = 0.05.

**Figure 3:**
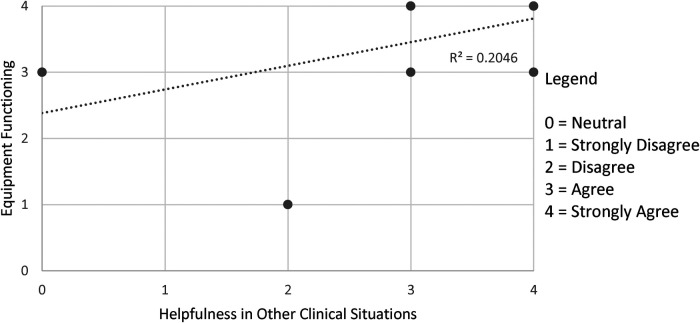
Equipment functioning and helpfulness in clinical situations. *Note.* The data displayed were from the statements “The equipment functioned as expected” and “I believe that this type of telemedicine approach would be helpful in other clinical situations”. There were 11 pairs data points submitted, repeated responses were not displayed. *r* = 0.759, *α* = 0.05.

Figures 1–3 demonstrate that the perceived utility of Peds-TECH is significantly correlated with expected audio-video capabilities and consistent functioning of the machinery supporting the program. The results indicate the functioning of the telemedicine machinery has a primary impact on physician's perceptions that the service benefits patient care. Namely, audio quality and the equipment functioning were positively correlated with the belief that a telemedicine approach similar to Peds-TECH would be helpful in other clinical situations as expected. Similarly, video quality was positively correlated with the belief that Peds-TECH was more effective than a phone consultation. Each of these findings were further explored during the interviews.

### Parent interviews

#### “Confident in the care environment”

The parent/caregiver experience of having their child treated with telemedicine through Peds-TECH was largely defined by trust in the program due to the recognized benefit of involving physicians from a specialized children's hospital in their child's care. The participants were all unfamiliar with the Peds-TECH program before their first interaction, but introduction of a connection to the remote hospital was met with feelings of assurance or confidence in the care being delivered. One participant noted that “I felt very confident in what was happening, especially when they introduced me to what they wanted to do with the link with [remote hospital] … I felt we were doing the right thing” [P0208_2021]. The remote hospital was associated with expertise and experience, leading parents/caregivers to feel confident in the care received. Participants also recognized the benefits of Peds-TECH through their own observations of the care process.

#### “Forever grateful”

Participants expressed gratitude for the use of telemedicine in their child's care. Gratitude was centred on two primary factors, the first being the connection to expertise and the second being the positive outcomes experienced by their child. As one participant said “I’m forever grateful for that technology, my son would not be here without it. And I said at 100% certainty he would not. [P0510_2021].” This participant was thankful for the program and supportive of more children benefiting from this type of care process based on their own positive experiences. Participants were aware that Peds-TECH allowed them to be connected to specialist pediatric care in a way that was unique and appreciated the collaborative approach of the program. The outcome of the care experience also had an impact on expressions of gratitude. None of the participants interviewed communicated any adverse events or outcomes as a result of their child's treatment through Peds-TECH. Participants expressed that they were grateful for or happy with the program because of the outcomes for their child.

#### “The experience of being transferred”

All children of the parents/caregivers interviewed were transferred to the remote hospital from a local hospital site following their experience with Peds-TECH. For some participants, Peds-TECH was perceived as aiding the transfer of care between sites because the providers were already familiar with the patient upon their arrival at the remote hospital. This was discussed distinctly from the other observed benefits of the program, as it was experienced after the telemedicine interaction. Discussing their experience, one participant said, “ …eventually a team came to bring him to [remote hospital] by ambulance. But it just- it felt like it was a good experience because they had that [program]. [P0409_2021].” By connecting with the team at the remote hospital before arriving at the hospital, there was a sense of familiarity during the trajectory of the care especially in the transition of care between sites.

### Physician interviews

#### “Providing visual perspective”

The ability of the remote physicians to see the patients facilitated the process of providing recommendations for care and aided communication during the resuscitation process. Participants from the remote hospital found that using Peds-TECH allowed them to understand the patients' condition without the need to make an assessment based on a verbal description alone. It also enabled them to observe the procedure being conducted by the local physicians and plan for the care of the patient upon arrival to their site. One participant said:

I found that it's very helpful for us to have a clearer video communication, prior to the patient arriving at our hospital … I think it allows us to, number one, immediately manage the situation whatever the child is experiencing, but also more than that let us plan for the child's arrival and eventual disposition. [P0712_2021].

The visual aspect of the program also benefitted the local physicians' communication by reducing the need to describe vital signs and the condition of the patient in terms of changes in colour, crying, and other physical characteristics. The visual component of the program communicated the level of urgency and changes in the disposition of the patient without a need for continual description. By minimizing the need for ongoing requests for vital signs, the visual connection gave participants from both sites more time to discuss other aspects of care. As stated by one participant:

I found that with a telephone experience I had to repeat the same information a few times in order for the team to kind of grasp the level of acuity that I was trying to relay and explain to them, or paint that picture. With a video it was quite apparent the level of acuity and the level of urgency that we were dealing with and so I found that the experience was much more smooth. [P0912_2021].

The visual aspect of telemedicine also enhanced communication between teams. For local physicians, it allowed them to be better understood and feel that they were “on the same page” as the remote physicians when discussing the patient, especially when it came to communicating the acuity of the patient.

#### “Institutional understanding”

Participants consistently discussed the resources available to them in the context of their broader experiences with telemedicine. Participants from the local hospital discussed limitations in personnel and specialized equipment that they often require when managing a resuscitation. A perceived lack of knowledge from the remote physicians about resources available at the local site contributed to concerns about the ability to complete all suggested procedures and led to some strain on the interaction. This was highlighted in comments such as:

… When you’re in a resuscitation situation, I don’t always- especially with Covid- we don’t always have the nurses like the [remote hospital] pediatric team. They’ve got a pharmacist there. They have a zillion nurses. They have so much more services available and we have so little compared to them. [P1801_2022].

Participants from both the local and remote hospitals expressed concern about the potential for Peds-TECH to become time-consuming. Although this was not the experience of most participants, it was a commonly shared concern that centred primarily on the availability of personnel to manage patients while the interaction was ongoing. As described by one participant:

[The program impacted patient care and outcomes] Positively for the patients over there. Unfortunately, sometimes negatively for the patients at my site … Well, it takes me out right? So, during that time that I’m helping the patient in the other site I’m not seeing patients at my site … And that negatively impacts the care I'm providing at my site. [P0612_2021].

The lack of open communication about resources underpinned the interactions between providers. Availability of staff was a common concern for both local and remote hospitals although there was a perception that the remote hospital had many more resources and may lack understanding about the practicalities within the local hospital.

#### “Communicating expectations”

One participant reported a very negative encounter with Peds-TECH, which they described as an “epic disaster”. Although the technology itself worked without issues throughout the encounter, the communication and collaboration with the other team was unfavorable in the view of the participant. Collaboration during this encounter was described as condescending and was defined by a lack of understanding and respectful communication. In addition to this, the participant did not receive the type of support they asked for or expected from the service.

Despite having two encounters with Peds-TECH that were positive in nature, the participant stated that they would hesitate to use telemedicine in the future, only using it when absolutely essential. This sentiment was not shared by any of the other participants, highlighting the importance of cordial communication, a supportive environment, and collaboration in creating willingness to engage with the program.

### Integration of findings

**Figure 4:**
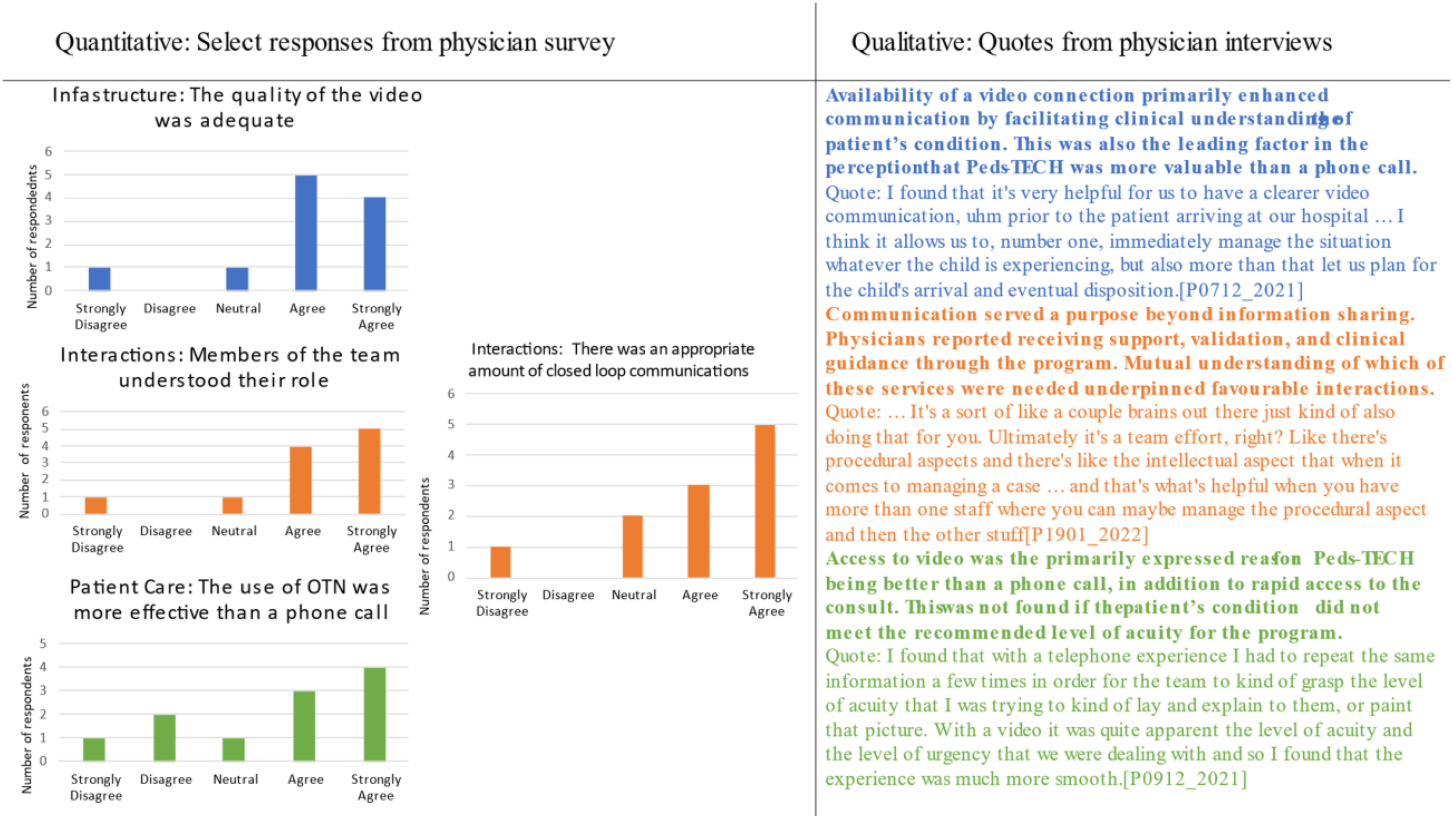
Joint display of qualitative and quantitative results from physicians.

Three primary concepts connected the quantitative and qualitative findings: (1) the visual component of the program, (2) interactions between providers, and (3) perception that the program was more effective than phone consultation. Examples of each concept can be seen in Figure 4. Each of these concepts was closely related in the participants’ overall perception of Peds-TECH interactions. Availability of a video connection enhanced communication by facilitating clinical understanding of the patient's condition. This was also the leading factor in the perception that Peds-TECH was more valuable than a phone call. 81.8% of survey respondents agreed or strongly agreed that the quality of video was adequate.

Interactions between providers went beyond information sharing, whereby local physicians reported receiving support, validation, and clinical guidance through the program. Mutual understanding of which of these functions was needed underpinned favourable interactions. 81.8% of survey respondents agreed or strongly agreed that members of the team understood their role during interactions, whereas 72.7% agreed or strongly agreed that there was an appropriate amount of closed-loop communications.

Finally, access to video was the primarily expressed reason for Peds-TECH being better than a phone consultation, in addition to rapid access to the consulting physician. This perception was not found in cases when the patient's condition did not meet the recommended acuity for the program, likely due to video not being deemed necessary for the consultation and concerns about the time spent on the consult for a non-critical case. 63.6% of participants agreed or strongly agreed that the program was more effective than a phone call, whereas 18.1% disagreed with the statement.

## Discussion

### Interpretation of findings

This pilot study reports on a wide range of perceived benefits of the Peds-TECH program and highlights operational challenges that could be addressed systematically. It was noted that the program had particular benefit because of its application in a pediatric patient population. Visualization of the patient is especially important in this population because patients are often unable to communicate how they feel and are prone to deteriorating more quickly than their adult counterparts (12). Additionally, the visual component improved the efficiency of communication by reducing the amount of time spent asking for and providing updates on visual cues and vital signs. Barriers and facilitators had a minimal impact on willingness to utilize Peds-TECH; rather they impacted the quality and effectiveness of each experience with the program. Lack of institutional understanding regarding available resources (e.g., staffing, nurse experience with pediatrics, patient flow pressures, etc.) was a barrier to optimal experiences with the program for physicians. Lack of understanding about expectations for the consultation, such as whether procedural guidance or another form of support managing the patient was needed, was a barrier to future use of the program for physicians and was discussed in conjunction with communication challenges. Clear and respectful communications were key facilitators of the telemedicine process.

Parents and caregivers expressed acceptance of and gratitude for Peds-TECH. Upon recognition of the severity of their children's conditions, parents/caregivers expressed gratitude for the opportunity to be immediately connected to specialized care without hesitation about the use of Peds-TECH. This was largely due to the reputation of the remote hospital as the leading children's hospital in the region, which reflected a pre-established sense of trust within the parents/caregivers. Parents/caregivers did not express concerns about the care delivered through the program owing to the sense that use of Peds-TECH was a routine method of care delivery and the confidence of the local physicians. For many parents/caregivers, Peds-TECH was not described as a central component of their care experience. Focus was on the care that was being given but not on the means of delivery. In spite of this, parents/caregivers recognized benefits of Peds-TECH as it related to their child's ability to quickly be seen by specialists. The perceptions of parents regarding use of Peds-TECH were initially influenced by their existing trust in the local and remote hospitals, followed by the observations and outcomes of their first-hand experience. Methods used in the study are feasible for a large-scale study but the addition of participation incentives is needed in order to increase successful participant recruitment.

### Comparison to previous studies

The literature largely supports use of telemedicine as a feasible and effective method to deliver care, but the application of and extent of use of telemedicine in different settings necessitates specialty-specific studies (13–15). Additionally, there is a paucity of information surrounding the user-experience or perceived effectiveness of tele-resuscitation programs in the pediatric population (16, 17). Sauers-Ford et al. (17) was the only article that provided an extensive evaluation of a pediatric telemedicine program in the ED. Beliefs about telemedicine being too time consuming, frustrating to use, and resulting in no changes to care plans were identified as barriers to physician uptake of telemedicine (6). Although concerns about time required for telemedicine were expressed in this study, it was not a barrier to use of the program nor were the other barriers identified in the literature.

There was no consensus in the literature regarding parent/caregiver perceptions of telemedicine use in their child's care. Some literature demonstrates a strong preference for face-to-face care in the context of pediatric rheumatology and home-based early intervention for developmental delays (18, 19). Alternatively, McConnochie et al. (20) found that parents were accepting of telemedicine, expressing gratitude for the service in the context of school and child-based telemedicine for acute childhood illness. The findings suggest that parent/caregiver acceptance of telemedicine may be related to the level of care being provided, whereby acceptance is high in acute or emergency contexts and lower in non-acute contexts.

### Clinical implications

The findings suggest that a telemedicine program has utility and acceptance among parents/caregivers and physicians for the treatment of critically ill pediatric patients in the ED. When using Peds-TECH, neither physicians nor parents/caregivers expressed concerns or hesitation about the care being delivered. Once the program was used, the benefits were rapidly recognized and highly valued by both parents/caregivers and physicians, including rapid connection to sub-specialized care and enhanced communication between remote and local service providers. This indicates that implementation of telemedicine programs may be welcomed by both populations but requires initial exposure.

### Research implications

Future research should seek to understand the benefits, limitations, and implementation of telemedicine programs within emergency pediatrics more extensively and across the continuum of care. Additional areas for further research include: exploration of the role of telemedicine use in outcomes of transfer, the relationship between patient acuity and acceptance of telemedicine among parents and caregivers, and influence on care quality, outcomes, perceptions, and cost of care.

### Strengths and limitations

The study has many strengths including data triangulation through the use of a survey and interview, use of semi-structured interviews to understand the parent/caregiver and physician perspective, and use of a mixed-methods approach. The study is not without limitations. A limitation is that the small sample size and resultant self-selection bias that may limit the transferability of results to other settings or other subspecialties. Limited response rate was addressed during recruitment by engaging in two to three rounds for recruitment for each participant group and expanding criteria to include all individuals (parents/caregivers and physicians) that had engaged with Peds-TECH since initiation of the program. Use of participation incentives may have aided recruitment efforts. The exclusive focus on physicians and parent/caregivers also limits understanding of interprofessional team perspectives that may have been gained by including allied health professionals in the study population. Recall bias is another potential limitation of the study, given that cases occurred over the course of multiple years. To mitigate recall bias, surveys were sent to physicians within 1 week following a given case; however, interviews were conducted several months after a case, ranging from 2 to 6 + months based on who was available for interview (21)*.* Additionally, all participants were invited to participate in interviews in order of recency and frequency of use to minimize the recall period (21).

Of the 11 survey respondents, one was an outlier that experienced issues that consistently impacted their interaction with the machinery across each category. Unfortunately, this participant did not fill the open-ended sections in their survey, meaning that there was no ability to elaborate on the issues that occurred during their interaction with the program. It can be assumed that either the participant experienced a case in which Peds-TECH should not have been used and a phone call would have been adequate (e.g., there was no need to visualize the patient or complexity did not meet the CTAS-1 or 2 requirement) or that the program was completely dysfunctional for the participant (e.g., neither the audio or video systems connected or a call could not be placed).

## Conclusion

This research findings addressed knowledge gaps regarding the perceived effectiveness of a pediatric tele-resuscitation program as well as implementation barriers and facilitators. Within the study population, parents/caregivers and physicians had positive perceptions of telemedicine for emergency pediatric care. Facilitating institutional understanding and expectations of the consultation may address barriers identified within the physician group and improve the experience of utilizing telemedicine. The methods utilized in this pilot study are feasible to be implemented in a larger scale study, with the addition of participation incentives to aid recruitment. A larger study is needed to validate the findings of this pilot study. More research is needed to understand the long-term clinical and economic impacts of Peds-TECH program and other similar digital innovations occurring in the similar healthcare environment.

## Data Availability

The raw data supporting the conclusions of this article will be made available by the authors, without undue reservation.
