# Supplementary material for: Examining perceptions of a telemedicine network for pediatric emergency medicine: a mixed-methods pilot study
Source: Front Digit Health. 2023 May 26;5:1181059. doi: 10.3389/fdgth.2023.1181059 (PMC10251235; doi:10.3389/fdgth.2023.1181059)
Supplement: Supplementary file 1 [file Datasheet1.pdf]

Appendix A: Health Professionals' Survey and Interview Guides

Email sent with survey link:

Subject line: Pediatric Teleresuscitation Program (Peds-TECH) Survey

Hello Dr. [Name],

I am emailing you regarding the case of pediatric teleresuscitation you recently completed through OTN. As you know, the teleresuscitation program is part of a larger initiative to evaluate and continually improve the teleresuscitation process through a project called Peds-TECH – Pediatric Telemedicine Connecting Hospitals. Through this project we would like to hear your feedback through this survey about the most recent case of pediatric teleresuscitation that you were involved in, in order to make changes and improvements to the process. Feel free to contact me with any questions and thank you for your participation. Here is the link to the survey:

[survey link]

Best,

[Name]

Section A: Case Description

Q1 Give a short provisional diagnosis of the patient. Please include information about the initial impression, and/or diagnosis reached after the tele-resus. Please include any specific interventions/treatments given as a result of the tele-resus.

Q2 What was your role in the case?

▼ Physician (1), Nurse (2), Other

Q3 Please highlight in what way(s) you feel the tele-resuscitation was better or worse that a phone call.

Q4 Through which organization/site were you involved with this particular case?

▼ MCH (1), SCS (2), WHS (3), GNG (3), DMH (4), PCG (5)

Section B: Infrastructure

Q1 Please comment on the infrastructure.

	Strongly Disagree (1)	Disagree (2)	Neutral (3)	Agree (4)	Strongly agree (5)
The quality of audio was adequate. (1)	○	○	○	○	○
The quality of video was adequate. (2)	○	○	○	○	○
It was easy to communicate over OTN. (3)	○	○	○	○	○
The communication was adequate to provide patient care. (4)	○	○	○	○	○
The equipment functioned as expected. (5)	○	○	○	○	○

Q2 Please provide any feedback or suggestions you have regarding the activation, equipment or connection.

Section C: Interactions

Q1 Please comment on your interactions with the teams at both sites.

	Strongly Disagree (1)	Disagree (2)	Neutral (3)	Agree (4)	Strongly agree (5)
Decision making was collaborative. (1)	○	○	○	○	○
Decision making was timely. (2)	○	○	○	○	○
It was easy to understand each other through the two way communications. (3)	○	○	○	○	○
Members of the team understood their roles. (4)	○	○	○	○	○
There was an appropriate amount of closed loop communications. (5)	○	○	○	○	○

Q2 Please provide any feedback or suggestions you have regarding the interaction of the teams and communication.

Section D: Patient Care
Q1 Please comment on the care that was provided to the patient through the tele-resuscitation case you were involved with.

	Strongly Disagree (1)	Disagree (2)	Neutral (3)	Agree (4)	Strongly agree (5)
The care of the patient was enhanced through the use of the tele-resuscitation process. (1)	○	○	○	○	○
The patient outcome was improved through this interaction. (2)	○	○	○	○	○
The use of OTN was more effective that a phone call (e.g. direct telephone consultation). (3)	○	○	○	○	○
The patient transfer of care was improved by the interaction. (4)	○	○	○	○	○
I would recommend this type of tele-resuscitation interaction to my colleagues. (5)	○	○	○	○	○
I believe that this type of telemedicine approach would be helpful in other clinical situations. (6)	○	○	○	○	○

Q2 Please provide us with any feedback or suggestions you have regarding patient care through tele-resuscitation
.Section E: Summary
Q1 Please provide us with any further information you believe is relevant to the case and process that we have neglected to ask. Also, if you have ideas for improvements to the process please provide them below.

Parent/Caregiver Interview Guide

Hello, my name is [name]. I am working with the Pediatric Tele-resuscitation project at Niagara Health. I am contacting you because your son or daughter was treated using the telemedicine technology and I would like to speak to you about your experience. Your participation is completely voluntary. You can respond to whichever questions you feel comfortable with and end the interview at any time. Is now a good time?

I would like to re-iterate that you can choose to end the interview at any time. The interview will take approximately 30 minutes of your time. Your responses are confidential and will not be associated with any identifiers for you or your child. Do you have any questions? If you are ready, we will proceed with the interview.

1. What did you witness in the process of care?
    a. Can you describe the care process?
    b. What did you think went well? Poorly?
2. What was your perception of the use of technology in your child's care?
3. How did the healthcare providers continue to inform you while engaging with the technology? How were you engaged in the process?
4. In terms of the interactions between the healthcare providers, is there anything you think could have been improved or changed?
5. Is there anything other part of your experience you would like to comment on?

Thank you for your time. If you have any questions moving forward feel free to contact [name] (Co-Investigator) at [email address] or [name] (Principal Investigator) at [email address].

Physician Interview Guide

Hello, my name is [name]. I am working with the Pediatric Teleresuscitation project and would like to speak to you about your experience with the OTN technology being used in the

emergency department. Throughout the interview I will refer to your general experience but if you would like to speak about a particular case feel free to do so. Your responses will not be associated with your name. Your participation is voluntary, you can skip any questions you don't want to answer and terminate the interview at any point.

Technology

1. Can you please tell me about your experience using the telemedicine service?
   a. How did the service impact the way you were able to provide care to the patient?
2. What did you expect when you used the telemedicine service? Were these expectations met? Why or why not? (i.e. how much time did you expect it to take)
   a. Did your expectations change after having used the service? If so, how?
   b. Had you provided care through telemedicine in other care settings before your first case with this service?
3. Did you experience any technological issues with the telemedicine service – if so what were those? How did they impact your thoughts about the service? Willingness to continue using the technology?
4. Did you experience non-technological issues when placing or receiving calls, for example getting connected to the appropriate person at the other site? How did this impact your thoughts about the service?

Perception of Care

1. How did you find the telemedicine service impacted the care process and patient outcomes?
2. Do you feel that the use of telemedicine service disadvantaged any health-related processes (e.g. was it too time consuming, did it impede the workflow, etc)? How?

3. How does the telemedicine compare to other methods of consultation, such as a phone call that you might have used in the past?
4. Given that the goal of the telemedicine service is to connect physicians across sites to ensure patients can be stabilized and supported in times of life saving care requirements. In your perspective, how has the use of telemedicine service helped to achieve this goal, or not?
5. Under what conditions do you think the program is most valuable, if at all?
    a. What types of patient conditions of acuity levels should it be used for in the ED?

Leadership, Teamwork, and Collaboration

1. Please describe the process of establishing leadership and collaborative care processes during the case.
2. How would you describe your interactions with the team at the other end of the call?
3. What was the team dynamic? Please describe any challenges or barriers to communication or collaboration that you experienced.

Overall Experience

1. Can you think of any additional features that would enhance your overall experience with the telemedicine service?
2. Would you consider using this particular telemedicine service moving forward?
3. Is there anything else about your experience that you would like to comment on?
